# Genome-wide linkage scan on estimated breeding values for a quantitative trait

**DOI:** 10.1186/1471-2156-4-S1-S61

**Published:** 2003-12-31

**Authors:** Delilah Zabaneh, Ian J Mackay

**Affiliations:** 1 Oxagen Limited, 91 Milton Park, Abingdon, Oxon, United Kingdom

## Abstract

**Background:**

A genome-wide linkage scan was performed on Replicate 1 of the simulated data for fasting triglyceride levels. The aim of this study was to implement mixed-model methodology to estimate breeding values for each individual for this trait and to assess the merit of these breeding values in linkage analysis. These breeding values utilize all the pedigree information, and the genetic and phenotypic correlations with other measured traits across the two cohorts. A genome-wide linkage scan was run on both the new breeding value traits and the original traits.

**Results:**

Using breeding values, a maximum LOD of 7.78 was found on chromosome 5 at a position very close to a gene underlying the triglyceride levels. This effect was not detected using the original trait.

**Conclusion:**

The results imply that estimating breeding values may be a suitable method of deriving traits for use in genome-wide scans.

## Background

The best linear unbiased prediction (BLUP) of the breeding value of an individual for a quantitative trait can be calculated by taking into account the genetic and environmental covariances among all related individuals and across all correlated traits [1,2]. Data from fixed effects such as sex or population can also be incorporated. This methodology has been the basis of many national and international animal breeding programs, where in its most complete implementation it is referred to as the animal model [3].

Here we explore the merit of using BLUP to generate breeding values for input into a genome scan. Our motivation is that frequently in human genetic analysis there is a primary trait of interest together with a number of covariates that may also be heritable. To improve the precision of measurement of the primary trait, we wish to remove the effect of any environmental covariation with the other traits, but include the effect of any genetic covariation. This is in essence what the animal model achieves.

For purposes of illustration, we have used fasting triglyceride level as our primary trait.

## Methods

We chose to analyze Replicate 1 of the complete simulated data sets without any knowledge of the underlying simulation model or the location of the trait loci.

### Pedigrees

The original number of pedigrees from Replicate 1 (after combining the original cohort and the offspring cohort) was 330 families. These comprised 4690 individuals with an average family size of 14, ranging between 7 and 84 individuals. After creating nuclear families (for the genome scan), 1444 pedigrees comprising 5808 individuals were formed, with a family average size of 4, ranging between 3 and 12 members.

### Estimating breeding values and (co)variances

The following account is taken from Mrode [4]. Similar descriptions are to be found in many outlines of animal breeding methods, for example Lynch and Walsh [5].

For a mixed model where all genetic variance is additive, the model is

**y_*i *_= X_*i*_b_*i *_+ Z_*i*_a_*i *_+ e_*i*_, **    (1)

where **y**_*i *_is a vector of observations on individuals, **b**_*i *_is a vector of fixed effects (sex and cohort in this case), **a**_*i *_is a vector of random additive genetic effects (breeding values), and **e**_*i *_is a vector of random residual effects. **X**_*i *_and **Z**_*i *_are incidence matrices relating the observations to the respective fixed and random effects, in all cases, subscript *i *relates to the i^th ^trait.

**a **and **b **are usually estimated simultaneously by solving Henderson's [6] mixed model equations (MME) for model (1):



where **X **and **Z **are as defined in equation (1). **G **is the additive genetic variance and covariance matrix for individual effects, **R **is the variance covariance matrix for residual effects, and **A **is the numerator relationship matrix that indicates the additive genetic relationship between each possible pair of individuals, for example in the absence of inbreeding, 1/2 for full sibs, 1/4 for grandparent-grandchild and 1.0 for an individual with him/herself (the diagonal elements of the matrix).

A model for a multivariate analysis for two traits could be written as:



The extension of model (3) for more than two traits follows the same pattern. Multivariate models have a corresponding increase in complexity for the MME, for more details see [4].

If **G **and **R **are unknown, these are also estimated from the MME. Here, all parameters were estimated utilizing the analytical gradient method of REML (restricted maximum likelihood) implemented in VCE [7]. The method can be extended to include common environmental effects and nonadditive genetic models, but this has not been attempted here.

Estimation of breeding values in this manner takes into account the presence and absence of data for all traits on all related individuals within a population. As a result, all individuals in the population have an estimated breeding value for all traits. In the extreme case of an individual with no observations and no relatives, the breeding value for a trait on that individual is the estimate of the population mean for that trait. With complete data on all individuals in a pedigree, estimated breeding values still differ from observed phenotype values: genetic and environmental correlations are used to include data on other traits measured on the same individual to improve precision, and genetic correlations similarly allow the inclusion of data from relatives. In the present context, aside from handling problems of sporadic missing observations, this means that breeding values for traits only measured in Cohort 1 can be estimated for individuals in Cohort 2, and vice versa.

### Traits

To fit limitations of time and software, principal component analyses (PCA) were used to construct new traits from the longitudinal data for each cohort using Genstat [8]. The new traits were created from the first principal component of the correlation matrix: the linear combination of the standardized original measurements that has maximum sample variance. For all new traits, the first principal component accounted for at least 90% of the variation, and loadings for each component trait were very similar so that the first principal component is almost equivalent to the mean of the measurements.

All longitudinal measurements were used from the Cohort 2 data for estimating these new PCA traits. Only a selection of such measurements were used from Cohort 1 to keep missing values in the two cohorts comparable: the implementation of PCA does not permit missing values in the component traits, and Cohort 1 had more missing values because measurements were taken over a much longer period of time. For Cohort 1, the original first measurement for fasting triglycerides was used instead of a PCA trait, as subsequent measurements have many missing values.

Although this procedure was applied to all traits with multiple measurements, due to software limitations only seven were taken forward for estimation of breeding values. These seven, three from Cohort 1 and four from Cohort 2, were selected based on the genetic and phenotypic correlations between the traits within each cohort. The aim was to select traits that contributed most in estimating genetic and phenotypic variance components for triglyceride, the primary trait of interest in this analysis. A summary of the chosen traits is in Table 1. Estimates of heritabilities, genetic, and residual (environmental) correlations, are in Table 2. Using these seven traits, two new breeding-value traits were therefore derived for all individuals: a triglyceride-Cohort 1 estimated breeding value (EBV) and a triglyceride-Cohort 2 EBV.

### Genome-wide linkage analysis

Five traits were analyzed separately in the genome-wide scan: triglyceride EBVs from Cohorts 1 and 2 (TG1_EBV and TG2_EBV, respectively), a simple average triglyceride trait from both cohorts in which every individual with a TG measurement on any occasion had a value (TG12_pooled), first PCA for Cohort 2 (TG2_PCA), and the original first TG measurement for Cohort 1 (TG1_original). These last three traits were included to compare with the EBV traits. Mega2 [9,10] was used to create nuclear families from the existing pedigrees and so reduce analysis time. Because many of the larger pedigrees were only connected by marriage, we presumed that any power loss would be minor.

**Figure 1:**
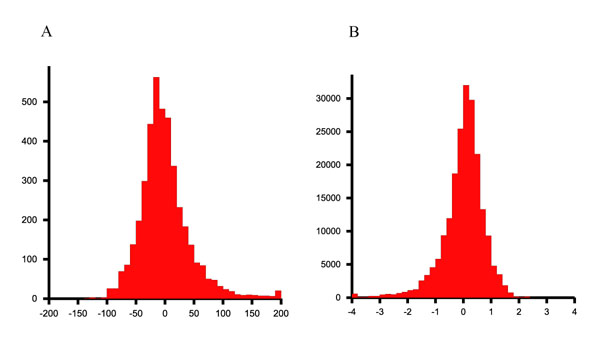
Distributions of EBV traits for Cohorts 1 and 2 for fasting triglyceride traits. A, Distribution of fasting triglycerides in Cohort 1 (first measurement) EBV trait. B, Distribution of fasting triglycerides in Cohort 2 (first PCA) EBV trait

Merlin-regress [11] was used for the genome-wide linkage analysis for the five traits. This is a new method based on the regression of the estimated IBD (identity by descent) sharing between relative pairs on the squared sums and squared differences of trait values of these pairs [11]. The variance components option in Merlin [12] was also used to confirm results for the breeding value traits because they were normally distributed (Figure 1).

## Results

Summaries of the maximum LOD scores from the regression method (using Merlin-regress) for the five genome-scans are in Table 3.

The pattern of results from the two cohorts was very similar for the triglyceride traits, therefore, only figures from Cohort 1 (original and EBV trait) are presented here.

**Figure 2:**
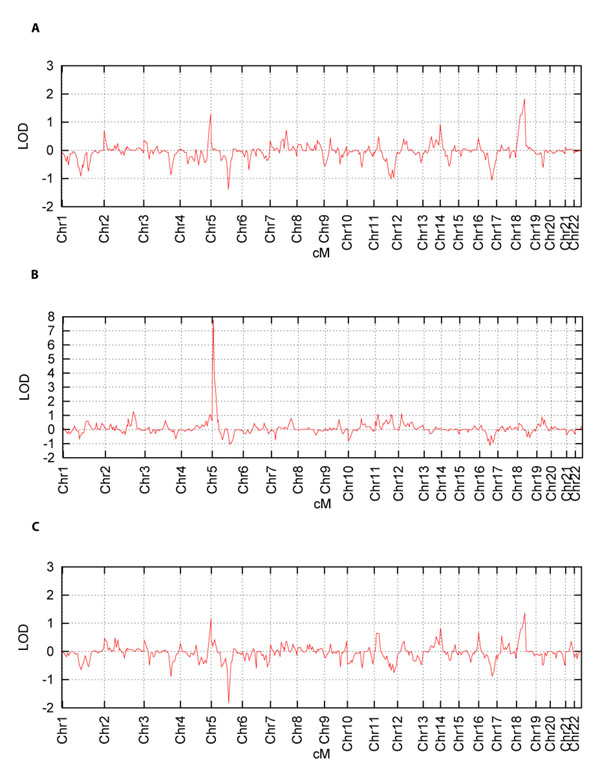
Genome scan LOD plots of fasting triglycerides in Cohort 1 (first measurement) (A), Cohort 1 (first measurement EBV) (B), and Cohorts 1 and 2 (pooled) (C)

Figure 2 shows plots of the LOD scores over the whole genome for TG1_original, TG1_EBV, and TG12_pooled, respectively.

## Discussion

The most notable results are the peak LODs of 7.78 and 6.86 on chromosome 5 at 8.22 cM, for TG1_EBV and TG2_EBV, respectively. These two peaks correspond very closely to the location of the gene *s3 *at 8.46 cM in this data set. It is noticeable that none of the three remaining traits provided evidence of linkage at this location: the maximum LODs on chromosome 5 for these being 0.85, 0.28, and 0.42 for TG1_original, TG2_PCA, and TG12_pooled, respectively.

Variance component analysis of the breeding values gave LODs of 5.12 and 4.50 for TG1_EBV and TG2_EBV, consistent with the results using Merlin-regress.

The derivation of an EBV for a single trait occurs without reference to marker data, and is designed only to improve the precision with which the additive genetic value of that trait is estimated. (Note however, in animal breeding EBVs are generally derived for multiple traits, or for indices across traits.) We speculate that the improved power we see here is primarily the result of the improved precision with which this additive value is estimated. Other multivariate methods used in linkage analysis can also include genetic and environmental correlations among traits, for example [13]. These methods generally attempt to improve power to detect QTL by searching for loci with pleiotropic effects. In human genetic studies however, there is often a single trait of primary interest. Other traits, although correlated to varying degrees both genetically and environmentally, are of less interest in their own right. In such circumstances, we believe the use of EBVs has much to offer and may be advantageous over an explicit search for pleiotropic QTL.

As can be seen in Table 3, the use of predicted breeding values also has the advantage of providing a trait for analysis for every individual. However, these breeding values will be correlated. Since the estimation of breeding value is independent of the marker data, we are hopeful that the consequence of this non-independence for type I error rate in the genome scan will be minimal, although this requires further study. The absence of large LODs at other locations in the genome scan lends some support to the type I error rate not being grossly increased.

To date, we have only applied this method to a single replicate. The analyses of many more replicates and traits are required before we can use this method with confidence.

## Conclusion

The estimation of breeding values using BLUP may be a suitable method of deriving traits for use in genome-wide scans. In particular, the method makes effective use of correlated traits and provides a simple framework for coping with missing data.

## Figures and Tables

**Table 1:** Description of traits used in the REML^A ^(co)variance component analysis, and estimation of BLUPs

**Trait**	**No. Measurements^B^**	**% Missing Observations**	**1^st ^PCA % Variation**
Cohort 1			
Alcohol (g/day)	2	7.42	100.00
Fasting glucose (mg/dl)	5	5.14	95.98
Fasting triglycerides (mg/dl)	1	15.17	NA
Cohort 2			
Alcohol (g/day)	5	6.73	100.00
Total cholesterol (mg/dl)	5	6.73	91.37
Fasting glucose (mg/dl)	5	6.73	94.20
Fasting triglyceride (mg/dl)	5	6.73	95.22

^A^See text for explanation. ^B^No. of measurements: number of consecutive measurements used for PCA. In the case of TG in Cohort 1, only one measurement was taken to keep the proportion of missing values roughly comparable across all traits from the same cohort.

**Table 2:** Estimates of heritabilities, genetic and residual correlations between traits from Cohorts 1 and 2

	**Cohort 2**				**Cohort 1**		
	
	**Alcohol**	**Cholesterol**	**Glucose**	**Triglyceride**	**Alcohol**	**Glucose**	**Triglyceride**
Cohort 2							
Alcohol^A^	**0.05 (0.02)**^B^	0.26 (0.08)	-0.20 (0.10)	0.25 (0.08)	0.10 (0.08)	-0.06 (0.25)	0.01 (0.09)
Cholesterol	-0.10 (0.04)	**0.71 (0.04)**	0.06 (0.02)	0.15 (0.02)	-0.19 (0.13)	0.07 (0.03)	0.07 (0.03)
Glucose	0.00 (0.03)	-0.04 (0.04)	**0.62 (0.03)**	0.48 (0.04)	0.12 (0.16)	0.98 (0.02)	0.52 (0.04)
Triglyceride	0.55 (0.02)	0.03 (0.03)	0.26 (0.04)	**0.40 (0.03)**	-0.48 (0.22)	0.58 (0.04)	0.94 (0.04)
Cohort 1							
Alcohol	0^C^	0	0	0	**0.04 (0.02)**	0.01 (0.18)	-0.65 (0.16)
Glucose	0	0	0	0	0.04 (0.05)	**0.65 (0.02)**	0.62 (0.04)
Triglyceride	0	0	0	0	0.56 (0.03)	-0.12 (0.07)	**0.64 (0.02)**

^A^These are the 1^st ^PCA of the traits described in Table 1, except for TG in Cohort 1, which is the original measurement; see text for details. ^B^Heritabilities (in bold) are on the diagonal, genetic correlations are above and residual correlations are below the diagonal. All parameters have their standard errors in parentheses. The multivariate mixed model implemented for the parameter estimates used traits across cohorts. ^C^The residual correlation between the two cohorts is equal to 0 by definition with the model fitted here.

**Table 3:** Maximum LOD scores for the four analyzed traits with their position on the chromosome

**Trait^A^**	**% Missing Values^B^**	**Chromosome**	**h^2C^**	**Max LOD**	**Position**
TG1_original	60.6	18	0.60	1.83	62.65
TG2_PCA	71.6	7	0.39	1.39	177.73
TG12_pooled	32.2	18	0.29	1.36	62.65
TG1_EBV	0.0^D^	**5**	**0.85**	**7.78**	**8.22**
TG2_EBV	0.0	**5**	**0.85**	**6.86**	**8.22**

^A^TG, triglyceride trait, number suffixing the trait indicates cohort, where TG12_pooled is a pooled trait from both cohorts; see text for details. EBV: estimated breeding value trait. ^B^Missing values: number of missing observations/total number of individuals (after forming nuclear families (5808 individuals)). ^C^h^2 ^= value input in the Merlin-regress program. For EBV traits, instead of using a h^2 ^of 1.0, a conservative value of 0.85 was assumed. ^D^The use of BLUP means that no EBV is missing.
